# Metagenomic insights into the urban–rural variation of antimicrobial resistance and pathogen reservoirs in untreated wastewater from central India

**DOI:** 10.3389/fmicb.2025.1722229

**Published:** 2026-02-11

**Authors:** Bailey Secker, Amit Nayak, Aliabbas A. Husain, Sudipti Arora, Aditi Nag, Sandeep K. Shrivastava, Andrew C. Singer, Rachel L. Gomes, Edward Acheampong, Saravana B. Chidambaram, Tarun Bhatnagar, Umashankar Vetrivel, Rajpal Singh Kashyap, Robert J. Atterbury, Adam M. Blanchard, Tanya M. Monaghan

**Affiliations:** 1 School of Veterinary Medicine and Science, University of Nottingham, Leicestershire, United Kingdom; 2 Advanced Research Centre, Dr G.M. Taori Central India Institute of Medical Sciences (CIIMS), Nagpur, Maharashtra, India; 3 Dr. B. Lal Institute of Biotechnology, Jaipur, India; 4 Centre for Innovation, Research & Development (CIRD), Dr. B. Lal Clinical Laboratory Pvt., Ltd., Jaipur, India; 5 United Kingdom Centre for Ecology and Hydrology, Wallingford, United Kingdom; 6 Food Water Waste Research Group, Faculty of Engineering, University of Nottingham, Nottingham, United Kingdom; 7 Department of Statistics and Actuarial Science, University of Ghana, Legon, Ghana; 8 Department of Pharmacology, JSS College of Pharmacy, JSS Academy of Higher Education & Research, Mysuru, India; 9 ICMR-National Institute of Epidemiology, Chennai, Tamil Nadu, India; 10 National Institute of Traditional Medicine, Indian Council of Medical Research, Belagavi, India; 11 Virology and Biotechnology/Bioinformatics Division, ICMR-National Institute for Research in Tuberculosis, Chennai, India; 12 NIHR Nottingham Biomedical Research Centre, Nottingham University Hospitals NHS Trust and the University of Nottingham, Nottingham, United Kingdom; 13 Nottingham Digestive Diseases Centre, Translational Medical Sciences, School of Medicine, University of Nottingham, Nottingham, United Kingdom

**Keywords:** antimicrobial resistance (AMR), India, low- and middle-income countries, One Health, resistome, wastewater metagenomics, wastewater surveillance

## Abstract

**Introduction:**

Rapid and scalable surveillance of antimicrobial resistance (AMR) is urgently needed in resource-constrained countries where routine monitoring is limited. Wastewater-based metagenomics offers a potential solution for early detection and geographic mapping of AMR.

**Methods:**

We conducted a retrospective DNA shotgun metagenomic analysis of untreated wastewater collected across Nagpur, India (February–April 2021). A total of 422 grab samples were pooled into 138 composite samples from 10 urban zones and rural catchments. The bacterial microbiota and resistome were profiled, and urban–rural patterns were compared using diversity metrics and correlation analyses.

**Results:**

Across all samples, 871 bacterial genera were detected, dominated by Proteobacteria, with frequent presence of *Pseudomonas*, *Acinetobacter*, *Aeromonas*, *Acidovorax* and *Bacteroides*. Beta diversity revealed statistically significant but subtle urban–rural compositional shifts. Of 33 globally important pathogens examined, 13 were detected at generally low relative abundance (<1%). *Vibrio cholerae* appeared in one sample, while *Aeromonas* spp. were most prevalent. Seven pathogens occurred in ≥10% of samples, with *Aeromonas*, *Citrobacter*, and *Enterobacter* differing significantly between locations (*p* < 0.05). The resistome comprised 606 unique antimicrobial resistance genes (ARGs), dominated by drug/biocide efflux determinants, followed by macrolide-lincosamide-streptogramin B genes driven largely by 23S rRNA mutations. Carbapenemases (*blaNDM*, *blaKPC*) and colistin resistance (*mcr*) were detected at lower abundance. Correlation analyses linked *Pseudomonas* with *mexEF*/*emhABC* efflux and *copBCDRS* copper resistance operon, *Acinetobacter* with *oxa* and *dfrA*, and *Aeromonas* with *ctx*, *tetA*, *sul1*, *dfrB/F*, and *gyrA/parC*.

**Discussion:**

These findings show that wastewater metagenomics sensitively resolved clinically relevant pathogens and ARGs in an Indian urban–rural setting, capturing nuanced geographic structure. Integrating routine DNA metagenomics into One Health environmental surveillance could strengthen AMR early warning and guide interventions in resource-constrained contexts.

## 1 Introduction

The COVID-19 pandemic catalyzed global recognition of wastewater-based surveillance (WBS) as a powerful and cost-effective tool for tracking emerging and endemic infectious diseases at the population level (Thompson et al., 2020; Wade et al., 2022). Wastewater reflects pooled biological signals from entire communities, capturing pathogens shed by both symptomatic and asymptomatic individuals. This makes it particularly valuable in low- and middle-income countries (LMICs), where traditional surveillance and diagnostic systems may be limited or fragmented (Crits-Christoph et al., 2021; Hendriksen et al., 2019). While WBS initially gained prominence through its role in monitoring SARS-CoV-2, its broader utility extends to the detection of respiratory, gastrointestinal, zoonotic, and antimicrobial-resistant organisms (Asghar et al., 2014; Knight et al., 2021; Lamba et al., 2017).

India presents a compelling case for expanded environmental surveillance. The country faces a dual challenge of high infectious disease burden and a growing concern around escalating antimicrobial resistance (AMR), influenced by high antibiotic use in medicine and agriculture, alongside limitations in wastewater treatment infrastructure (Graham et al., 2019; Klein et al., 2018; Laxminarayan and Chaudhury, 2016). A recent systematic review highlights that the treatment of hospital wastewater fails to eliminate pathogens and AMR genes, resulting in hospitals representing a significant AMR environmental burden and public health risk (Amin et al., 2024). Despite increasing evidence of their diagnostic value and cost-effectiveness (Marais et al., 2023), investment in advanced technologies such as clinical and environmental metagenomics remains limited in many LMICs, including India. This slower uptake is not primarily a consequence of insufficient interest, but rather of structural challenges. These include fragmented or incomplete sewerage infrastructure, restricted laboratory and bioinformatics capacity, financial constraints, outdated or underdeveloped policy frameworks, limited recognition of utility within existing surveillance systems, and the considerable time and resources required to establish and sustain training in new methodologies (Getchell et al., 2024).

Environmental reservoirs, especially untreated urban and rural wastewater, play a critical role in the emergence, amplification, and dissemination of AMR. These reservoirs facilitate horizontal gene transfer and serve as convergence points for human, animal, and environmental microbiomes, making them a priority in One Health surveillance strategies (Huijbers et al., 2015; Larsson et al., 2018; Wellington et al., 2013). Despite this, comprehensive metagenomic datasets from Indian wastewater systems remain scarce, and the urban and rural perspective is lacking. Most previous studies have used culture-based or polymerase chain reaction (PCR)-targeted approaches, limiting insights into microbial diversity and resistome structure across different environments (Balkrishna et al., 2024; Rout et al., 2023; Taneja and Sharma, 2018).

We previously reported the first RNA-Seq-based analysis of untreated wastewater in India, conducted during the second wave of COVID-19. This study analyzed composite samples collected from urban and rural areas of Nagpur, Central India, and revealed extensive circulation of SARS-CoV-2, hepatitis C virus, and a range of zoonotic and enteric viruses, including chikungunya, rabies, and Jingmen tick virus (Stockdale et al., 2023). The study demonstrated the power of unbiased RNA metagenomics to detect unexpected viral threats, assess co-infections, and monitor pathogen distribution across diverse geographic settings.

Building on this platform, the present study applies DNA-based shotgun metagenomic sequencing to the same wastewater surveillance network. Our dataset comprises 138 composite samples spanning both urban and rural catchments, one of the largest of its kind in India, providing unprecedented resolution of bacterial communities and the antimicrobial resistome in this setting. Our objectives were to (1) assess the microbial composition and antimicrobial resistance gene (ARG) burden in urban versus rural wastewater catchments; (2) detect the presence and abundance of clinically important bacterial pathogens and resistance genes; and (3) explore correlations between key microbial taxa and ARGs. By combining viral and bacterial metagenomic data from the same community surveillance network, we aim to generate a more holistic understanding of public health threats and AMR risks in a major urban–rural region of central India. In doing so, we demonstrate the scalability and relevance of wastewater metagenomics for integrated infectious disease surveillance under a One Health framework in LMIC settings.

## 2 Materials and methods

### 2.1 Approvals for wastewater sample collection

As this was an environmental sampling study, no formal ethics were required from the respective institutions. However, official permissions for sample collection were taken from the Nagpur Municipal Corporation (NMC).

### 2.2 Study design

We conducted a retrospective cross-sectional DNA metagenomic analysis of untreated wastewater samples in Nagpur district, Maharashtra, during the second wave of the COVID-19 pandemic (3rd of February to 3rd April 2021). A total of 422 samples (1 L each) were collected using sterile plastic (HDPE) leak-proof containers from the main sewer drainage lines of 10 urban municipality zones and from open drains or surface water bodies in rural areas from Nagpur district. Sampling sites were selected in consultation with technical staff from the Nagpur Municipal Corporation (NMC) to identify primary sewer junctions receiving convergent flow from adjacent residential catchments, thereby ensuring that collected samples reflected mixed community effluent. Site selection additionally accounted for accessibility and operator safety under COVID-19 restrictions (Supplementary Table 1). Samples were collected by directly filling the containers to the 1 L mark. Each container was sanitized with 70% ethanol, labeled with zonal identifiers, and sealed in zip-lock bags. Sampling was carried out in the morning hours (07:30 and 12:00), when wastewater flow rates were expected to peak due to higher defecation frequency, as described by Heaton et al. (1992). All collections were performed under biosafety conditions in accordance with the Government of India standard operating procedures (Government of India, 2020).

The samples were immediately transferred under cold chain conditions (4 ± 2 °C) to the Research Department at the Central India Institute of Medical Sciences (CIIMS), using Thermocol boxes with ice packs. Under aseptic conditions, individual samples were pooled in equal volumes across geography and time to generate 138 composite samples comprising 110 composite samples from urban zones and 28 from rural areas. Pooling ensured that each composite sample was representative of a defined geographic catchment and minimized variability due to temporal or diurnal fluctuations in microbial load. In addition to reducing overall sequencing costs, pooling improved the likelihood of detecting low-abundance pathogens and antimicrobial resistance genes (ARGs) by concentrating signals from the same area. Composite samples were subsequently transported under cold chain to the Dr. B. Lal Institute of Biotechnology in Jaipur for further processing.

### 2.3 Sample collection, processing, and nucleic acid extraction

The samples were stored and processed at the Dr. B. Lal Institute of Biotechnology following protocols as previously described (Stockdale et al., 2023). In brief, after collection, all samples were stored at 4 °C for no longer than 24 h before pre-processing. Containers were sterilized by UV treatment for 30 min, followed by heat inactivation in a 70 °C sonicating water bath for 90 min. The samples were then brought to room temperature (21 °C) and subjected to a two-step filtration process: initial filtration through Whatman qualitative grade 40 paper, followed by vacuum filtration using a Millipore 0.45-μm membrane filter. Nucleic acids were precipitated by combining 50 mL of the filtrate with 0.9 g sodium chloride (NaCl) and 4 g polyethylene glycol (PEG) in a 50-mL Falcon tube. After dissolution, the mixture was centrifuged at 4 °C for 30 min at 5750 g. The supernatant was decanted, and the resulting pellet was re-suspended in the RNA/DNA Shield, provided in the ZYMOBIOMICS ™ kit. Nucleic acids were extracted using the ZymoBIOMICS MagBead DNA/RNA kit (R2136) according to the manufacturer’s instructions.

### 2.4 Library construction and sequencing

The quality of extracted DNA was assessed using a NanoDrop spectrophotometer and quantified using a Qubit Fluorometer (Thermo Fisher Scientific, United States). DNA libraries were prepared using the Illumina TruSeq® Nano DNA Library Preparation kit (Illumina, United States), with 100 ng of input DNA per sample. DNA was fragmented to an average size of approximately 350 bp following the manufacturer’s protocol. Library quality and fragment size distribution were evaluated using an Agilent Tapestation with high-sensitivity D1000 ScreenTape (Agilent Technologies, United States). Sequencing was performed on an Illumina HiSeq 2,500 platform using 2 × 150 bp paired-end reads, generating approximately 5 GB of raw data per sample. FASTQ files, quality checked and adaptor trimmed, were provided by Eurofins Genomics (Bengaluru, India).

### 2.5 Metagenomics analysis

Illumina paired-end reads were quality-assessed and trimmed to remove poor quality bases (−q 20) and short reads (−l 50) using FastQC (v0.12.1; Andrews, 2010) and FastP (v0.23.2; Chen et al., 2018), respectively. Host-derived sequences were filtered out using Hostile (v1.1.0; Constantinides et al., 2023) with a masked host reference database to enhance bacterial sequence retention. Microbial taxonomic profiling was performed using MetaPhlAn 4 (v4.1.1; Blanco-Míguez et al., 2023). ARGs were detected using AMR++ (v3.0.6; Bonin et al., 2023) with the MEGARes 3 database. Read counts were deduplicated and subjected to single-nucleotide polymorphism (SNP) verification to ensure ARG specificity. Relative abundance of ARGs was calculated from raw counts obtained from AMR++, and normalization was conducted using Cumulative Sum Scaling (CSS) implemented in the metagenomeSeq R package (v1.50.0; Paulson et al., 2013). Where required, relative abundance was calculated using the normalized counts. Coverage of specific genes was calculated by first identifying the length of each gene and the number of bases covered for each gene of interest using the deduplicated sequence alignment files from AMR++ (Bonin et al., 2023) and SAMtools v1.21 (Danecek et al., 2021), with secondary and low-quality alignments excluded. Gene-level abundance and presence were determined using ResistomeAnalyzer (Lakin et al., 2017) with default parameters. Resistance genes were only reported when read alignments met minimum identity requirements and covered a substantial proportion of the reference gene length (default ≥80% gene coverage), thereby excluding partial or low coverage matches arising from short conserved regions or mobile genetic element fragments. As the MEGARes 3 database contains multiple sequences for each gene, percentage coverage was calculated as (∑(bases covered) / ∑(gene length) * 100). Alignments failing to meet these thresholds were discarded, ensuring that reported resistance genes represent near full-length gene coverage, reducing inflation of ARG detection due to ambiguous or low-depth mapping (Supplementary Table 2).

### 2.6 Data and statistical analysis

All statistical analyses and data visualizations carried out in this study were conducted in R (v4.4.1). The continuous variables were compared using the independent samples t-test when the normality assumption is valid, as evaluated using the Shapiro–Wilk test, or the Wilcoxon rank-sum test otherwise. The specific test used is stated where appropriate. To characterize community structure and its determinants, we combined diversity, multivariate, and statistical association approaches: alpha diversity metrics quantified within-sample richness and evenness, multivariate analyses of community structure examined between-sample compositional differences, and correlation analysis assessed relationships between diversity, ordination scores, and relevant environmental or clinical variables. Each of these approaches is described briefly in the subsequent paragraphs.

Alpha diversity metrics (Shannon, Simpson, and species richness) and beta diversity (Bray–Curtis dissimilarity) were calculated using MetaPhlAn supporting scripts. Diversity analyses for ARGs were based on normalized counts and similarly assessed using Shannon, Simpson, and Bray–Curtis metrics calculated using the vegan package (v2.7–1; Oksanen et al., 2025).

Multivariate analyses of community structure were carried out using non-metric multidimensional scaling (NMDS), permutational multivariate analysis of variance (PERMANOVA), and permutational analysis of multivariate dispersion (PERMDISP). NMDS was performed using Bray–Curtis dissimilarity matrices, implemented via the metaMDS function in the vegan package. PERMANOVA was performed with 999 permutations using adonis2 to assess the differences in microbial and ARG community composition between urban and rural samples. Finally, PERMDISP, implemented via the betadisper function in the vegan package, was used to examine differences in multivariate dispersion among groups.

Spearman’s rank correlation analysis was performed to assess associations with the relative abundance of ARGs and bacterial genera present in at least 50% (69/138) of the metagenomic samples. Hierarchical clustering was subsequently performed using a distance matrix calculated as 1- Spearman correlation coefficient, with complete linkage.

Finally, the representative map of the Nagpur district, India, was produced using the Quantum Geographic Information System Software (QGIS) v3.40.7 (QGIS, 2025), using points with the longitude and latitude of the collection locations of the samples.

## 3 Results

**Figure 1:**
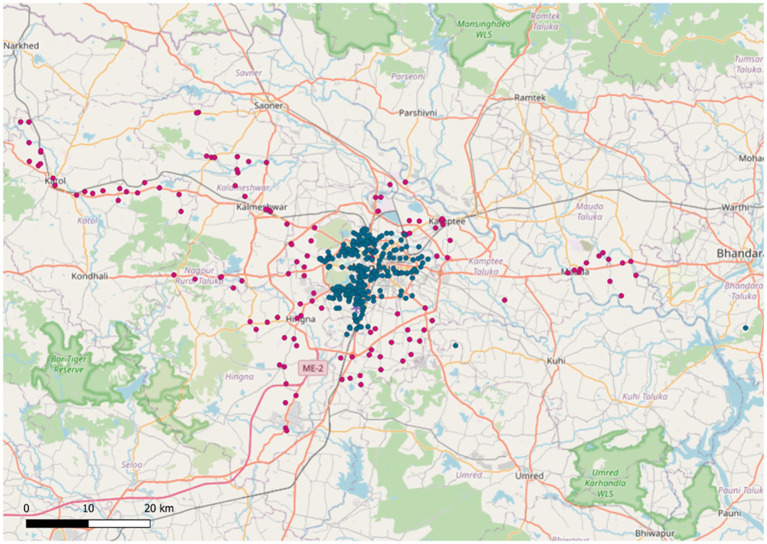
Map of the Nagpur district, central India, indicating geographical locations of sample collection sites contributing to composite samples produced using QGIS. Blue points denote urban sampling locations, while pink points denote rural sampling locations.

In this study, a retrospective DNA metagenomic survey was performed using 138 untreated composite wastewater samples collected from rural (*n* = 28) and urban (*n* = 110) areas of Nagpur, Central India (Figure 1).

### 3.1 Comparison of urban vs. rural samples

**Figure 2:**
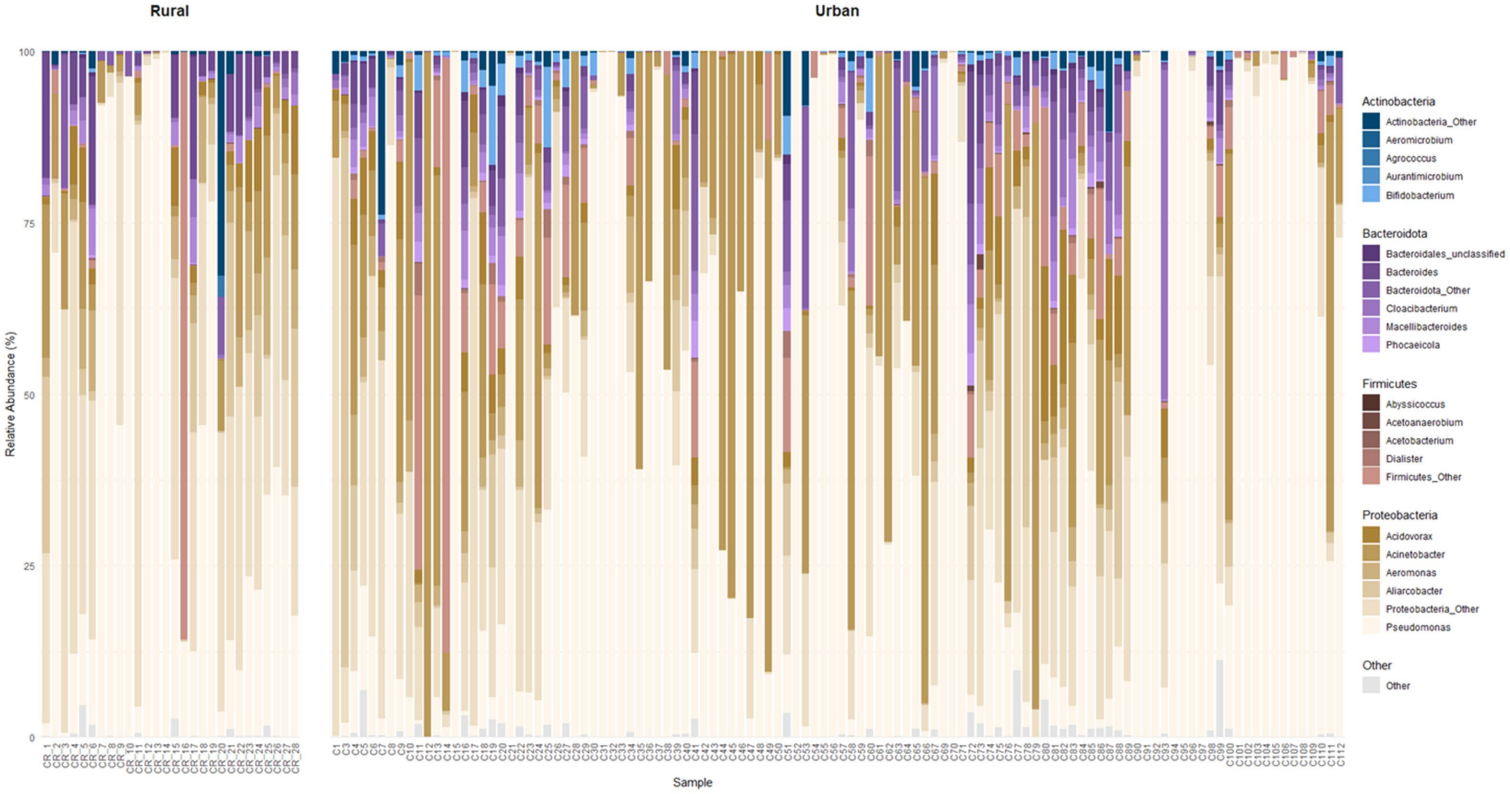
Relative abundance of the four phyla with the highest median abundance across all samples, together with the most abundant genera (by median abundance) within each phylum. Genera are color-coded according to phylum: shades of blue for Actinobacteria, purple for Bacteroidota, brown for Firmicutes, and beige for Proteobacteria. Genera belonging to all other phyla are shown in gray.

Shotgun metagenomic analysis identified 871 unique genera across all samples, with 97% of classified taxa belonging to the domain Bacteria. The majority of classified reads were assigned to four dominant phyla, shown as the median abundance and interquartile range (IQR): Actinobacteria (0.42%; 2.06%), Bacteroidota (3.68%; 12.8%), Firmicutes (0.65%; 4.90%), and Proteobacteria (91.9%; 22.1%) across all samples. Proteobacteria overwhelmingly dominated samples from both locations. Within Proteobacteria, the presence of *Pseudomonas* was prevalent across all samples, with a median (IQR) relative abundance of 34.4% (83.9%). Other frequently detected genera were *Acinetobacter*, *Aeromonas*, *Acidovorax,* and *Bacteroides* (Figure 2). No statistical differences were observed in alpha diversity between urban and rural samples (*p* = 0.6).

Beta diversity analysis revealed significant compositional differences between locations (PERMANOVA, *p* < 0.001). To assess whether this may be influenced by differences in within-group dispersion, we used the betadisper function from vegan. The results indicated a significant difference in dispersion between urban and rural groups (*p* < 0.01), suggesting that the observed PERMANOVA result should be interpreted with caution, as compositional differences may be confounded by heterogeneity in variance structure (Supplementary Figure 1).

### 3.2 Presence of potentially clinically relevant taxa in untreated wastewater samples

Globally, 33 bacterial pathogens have been implicated in 13.6% of all deaths and 56.2% of sepsis-related deaths in 2019 (Ikuta et al., 2022). Due to their relevance, metagenomic sequences were specifically interrogated for these 33 pathogens. Overall, 13/33 (39%) of the targeted pathogens were detected across the wastewater samples, although their abundance was generally low (Supplementary Table 3). When considering the ESKAPE group, all pathogens except *Staphylococcus aureus* were detected. *Vibrio cholerae* was also identified in a single sample. Despite the high prevalence of the *Pseudomonas* genus, *P. aeruginosa* was only identified in 6/138 samples.

**Figure 3:**
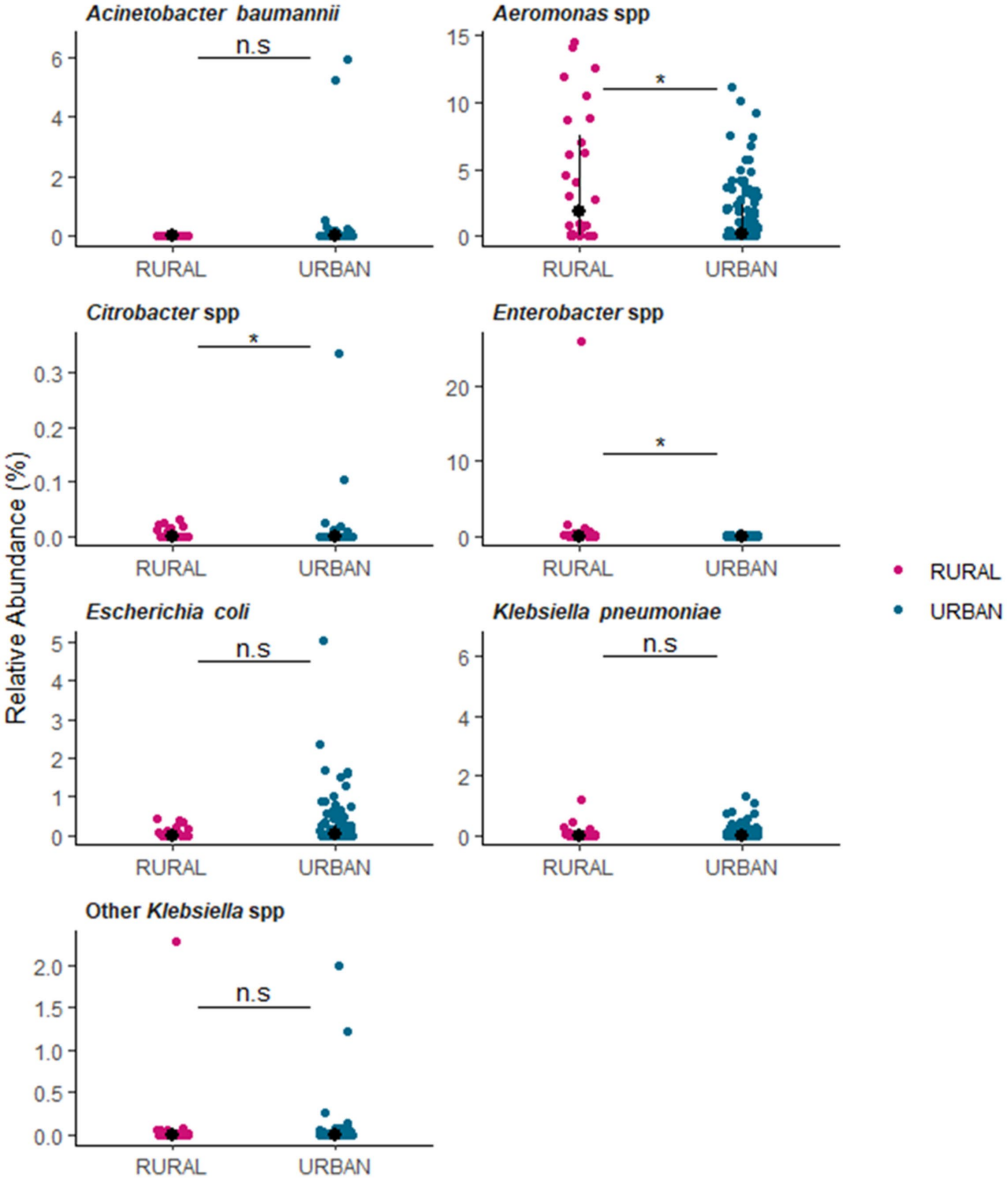
Relative abundance of clinically relevant human pathogens detected in at least 10% of rural and urban samples. Data are presented as median values with 25th and 75th percentiles. Statistical significance was assessed using the Wilcoxon rank-sum test with Benjamini–Hochberg correction (*p < 0.05).

To ensure robust statistical analysis, only pathogens present in at least 10% of samples were retained for further comparison. This threshold yielded seven pathogens for downstream analysis. Using the Wilcoxon rank-sum test with Benjamini–Hochberg correction, the comparison of the relative abundance showed no significant difference between urban and rural samples for *Escherichia coli*, *Acinetobacter baumannii*, *Klebsiella pneumoniae*, and other non-pneumoniae *Klebsiella*. However, *Aeromonas* spp., *Citrobacter* spp., and *Enterobacter* spp. demonstrated a significant difference between sampling sites (*p < 0.05; Figure 3).

### 3.3 Abundance and diversity of ARGs in environmental samples

A total of 606 unique ARGs were identified across all the samples, the most prevalent category of which was drug and biocide resistance (19.1%), primarily comprising genes encoding efflux pumps. This was followed by resistance to macrolide-lincosamide-streptogramin B (MLS) antibiotics, accounting for 17.6% of detected ARGs, which was largely due to mutations in the 23S rRNA gene (Supplementary Figure 2). Other clinically significant ARGs were also identified at lower abundance; this included carbapenemases such as *blaNDM* and *blaKPC* (3.5%) and colistin resistance from *mcr* (0.7%), although the coverage of these genes across all samples was low, 2.4 and 2.6%, respectively.

**Figure 4:**
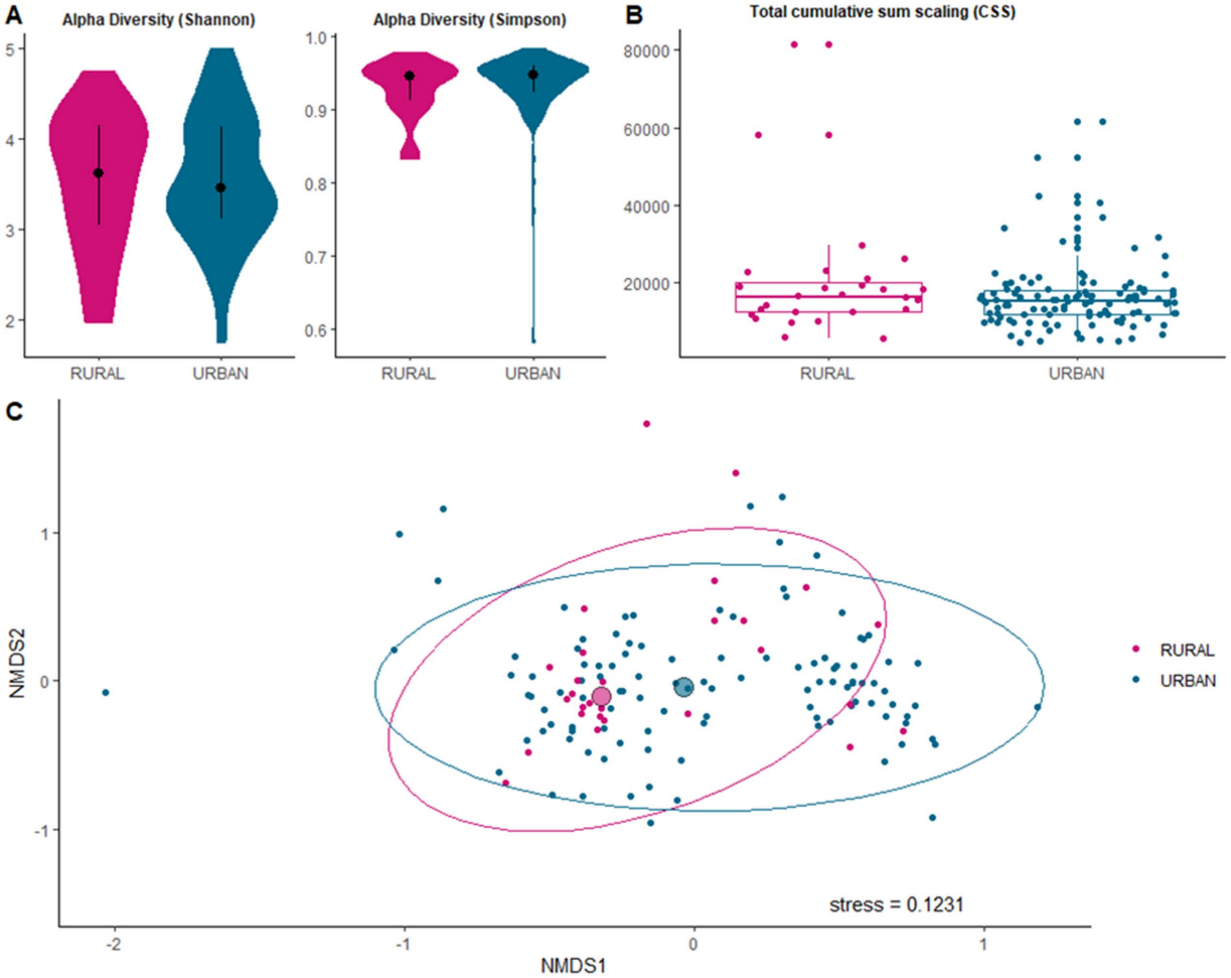
**(A)** Violin plot showing Shannon and Simpson alpha diversity metrics for ARG diversity in samples from rural and urban locations. Black point shows medians, with whiskers representing the 25th and 75th percentiles. **(B)** Box and whisker plots showing total antimicrobial resistance gene abundance per sample across both locations. **(C)** NMDS ordination plots of Bray–Curtis dissimilarity of the antimicrobial resistance gene composition in rural and urban samples. Each point represents an individual sample, with separation reflecting compositional differences. Larger circles denote median NMDS1 and NMDS2 values for each location. The stress value indicates the goodness-of-fit of the NMDS.

No significant difference was observed in the alpha diversity of ARGs between urban and rural samples (Shannon, *p* = 0.745; Simpson, *p* = 0.606; Figure 4A). ARG abundances were normalized using cumulative sum scaling (CSS) to account for variation in sequencing depth across samples. This normalization reduced compositional bias and enabled robust comparison between urban and rural wastewater communities. No statistically significant difference was observed in total ARG abundance between urban and rural sites (Figure 4B). However, there was a significant difference in the beta diversity (PERMANOVA, *p* < 0.01). This variation was unlikely to be driven by differences in within-group dispersion, as the PERMDISP result was not significant (*p* = 0.05; Figure 4C).

### 3.4 Relationship of ARGs and bacterial genera

**Figure 5:**
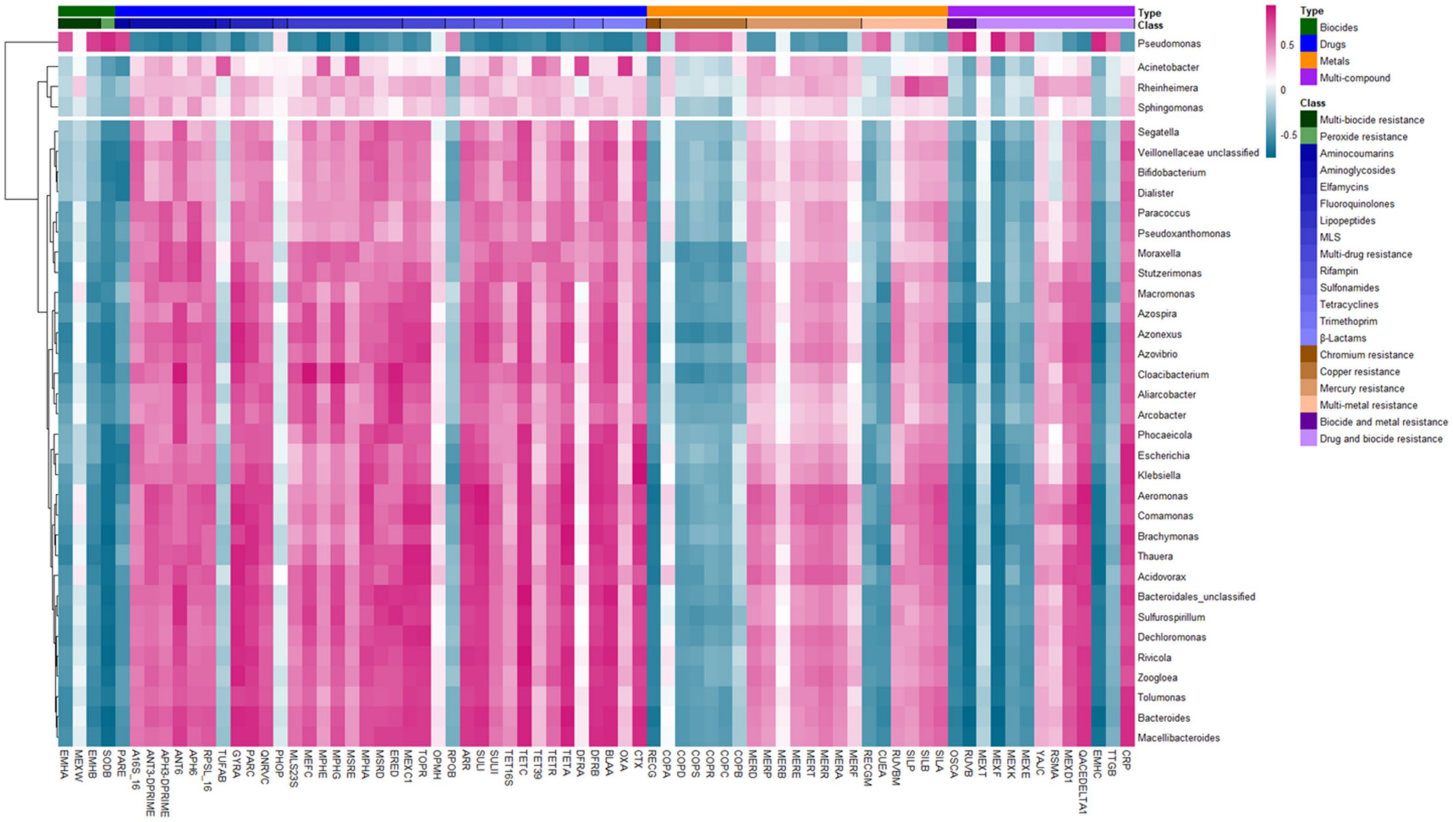
Heatmap of Spearman’s rank correlations between bacterial genera and antimicrobial resistance genes. Pink shading indicates positive correlations, while blue indicates negative correlations. Hierarchical clustering was applied to group bacterial genera based on their correlation profile. The color bar at the top denotes antimicrobial resistance gene classes as classified by the MEGARes database.

To investigate associations between ARGs and bacterial genera that were present in more than 50% of the samples, hierarchical clustering was used to group genera using Spearman’s correlation coefficient values (Figure 5). This analysis revealed three distinct clusters of genera based on their correlation with ARGs. The first cluster consisted solely of *Pseudomonas*. ARGs strongly associated with *Pseudomonas* were generally negatively correlated with other genera, suggesting a distinct resistome profile. Notably, *Pseudomonas* showed strong associations with efflux pump genes *mexEF* and *emhABC*, as well as the *copBCDRS* copper resistance operon, consistent with known resistance mechanisms in this genus. The second cluster contained three genera, notably *Acinetobacter*, which was strongly associated with resistance genes *oxa* (beta-lactamases) and *dfrA* (trimethoprim resistance). The third cluster comprised multiple genera, including *Aeromonas*, which were broadly associated with diverse ARGs across multiple classes, such as *ctx*, *tetA*, *sulI*, *dhrF/B*, and quinolone resistance determinants *gyrA/parC*.

## 4 Discussion

This study provides one of the most comprehensive DNA metagenomic analyses of untreated wastewater from both urban and rural areas in central India. By analyzing 138 composite samples across Nagpur district, a central transport hub with extensive human and goods movement, we deliver high-resolution insights into microbial composition and antimicrobial resistance gene (ARG) burden at the community level. Our findings demonstrate that wastewater grab samples can yield valuable information on the presence of ARGs and the gut-associated microbial composition of populations within the study site. When integrated with complementary data sources, such as clinical isolates or antibiotic usage records, or extended through longitudinal sampling, this approach could reveal trends and shifts over time, offering a foundation for improved understanding of population health and antimicrobial resistance dynamics in settings where formal diagnostic systems are under-resourced.

Bacterial antimicrobial resistance (AMR) is a major global health challenge, directly responsible for an estimated 1.14 million deaths in 2021. South Asia is projected to have the highest AMR-related mortality across all age groups by 2050 (Naghavi et al., 2024). The environment acts as an important reservoir for AMR, facilitating resistance transmission between humans, animals, and natural ecosystems (Larsson and Flach, 2022). Surveillance of untreated wastewater is therefore crucial for identifying clinically important pathogens and ARGs circulating within communities.

Multiple mechanisms contribute to the emergence, maintenance, and dissemination of antimicrobial resistance in wastewater environments. Selective pressure from residual antibiotics, even at sub-inhibitory concentrations, can maintain resistance genes and promote horizontal gene transfer (Gullberg et al., 2011). Co-selection by heavy metals and biocides, which are commonly present in wastewater, can indirectly maintain antibiotic resistance genes through genetic linkage on mobile elements (Baker-Austin et al., 2006). The high microbial density and taxonomic diversity of wastewater create optimal conditions for horizontal gene transfer via conjugation, transformation, and transduction, facilitated by mobile genetic elements including plasmids, integrons, insertion sequences, and transposons (von Wintersdorff et al., 2016). Differential inputs to wastewater systems further shape resistome composition: urban catchments receive substantial contributions from hospitals and healthcare facilities, where antibiotic selective pressure is intense (Berendonk et al., 2015; Lépesová et al., 2020; Verlicchi et al., 2012); rural catchments receive greater proportional inputs from agricultural sources, including livestock excreta containing veterinary antimicrobials and resistant commensal bacteria (He et al., 2020; Manyi-Loh et al., 2018); and both urban and rural systems receive domestic sewage containing human gut microbiota influenced by community antibiotic consumption (Bengtsson-Palme et al., 2016; Pärnänen et al., 2019; Pazda et al., 2019). These overlapping selection pressures and transmission pathways make wastewater a critical convergence point for resistance genes from multiple sources, underlining its importance for One Health surveillance (Hassoun-Kheir et al., 2020; Pruden et al., 2021).

Consistent with previous Indian studies, our results show a dominance of Proteobacteria across all samples, followed by Bacteroidota, Firmicutes, and Actinobacteria (Gawande et al., 2025; Marathe et al., 2019). The most abundant genera included *Pseudomonas*, *Acinetobacter*, *Aeromonas*, and *Acidovorax*, all associated with environmental and anthropogenic sources. This phylum-level structure mirrors shotgun metagenomic profiles from six hospitals across India, where Proteobacteria and Bacteroidota similarly dominated, with *Pseudomonas* and *Acinetobacter* leading depending on geography (Marathe et al., 2019). A hospital-based survey from Chennai further highlighted *Acinetobacter* dominance, alongside high prevalence of *sul1* and *mphE* conferring sulphonamide and macrolide resistance, respectively (Marathe et al., 2019).

In our dataset, *Pseudomonas* was uniquely abundant in both urban and rural settings, strongly associated with efflux pumps (*mexEF* and *emhABC*) and copper resistance operons (*copBCDRS*). These links highlight its dual role in metal tolerance and AMR, aligning with previous reports from wastewater and aquatic systems (Pal et al., 2015; Poole, 2001). *Acinetobacter* formed a separate cluster, associated with *oxa* and *dfrA* genes, reflecting its well-established multidrug resistance profile. A third, broader cluster including *Aeromonas* carried diverse ARGs, such as *ctx*, *tetA*, *sulI*, *dfrB/F*, and *gyrA/parC*, suggesting high potential for lateral gene transfer. *Aeromonas* spp. also showed significantly higher relative abundance in rural samples compared to urban samples (Mann–Whitney U-test, *p* < 0.05), potentially reflecting greater exposure to environmental water sources, agricultural runoff, and animal reservoirs characteristic of rural settings. *Aeromonas* species are ubiquitous in aquatic environments and are known to colonize agricultural systems (Janda and Abbott, 2010). Similarly, *Enterobacter* spp. showed significantly higher relative abundance in rural samples, likely owing to its role as an opportunistic pathogen in plants, animals, and humans, as such is readily identified in environmental samples such as wastewater (Davin-Regli et al., 2019). Conversely, *Citrobacter* spp. demonstrated significantly higher relative abundance in urban samples (*p* < 0.05), which may reflect inputs from healthcare facilities, as this organism is a known opportunistic nosocomial pathogen (Jabeen et al., 2023). These urban–rural differences in pathogen profiles suggest distinct transmission pathways and reservoir dynamics between the two settings.

Microbial alpha diversity did not differ between urban and rural locations, whereas beta diversity analyses indicated subtle but statistically significant differences in both community and resistome structures. These findings indicate that while overall ARG richness and evenness were similar across urban and rural catchments, the composition of resistance gene profiles differed significantly between locations. These patterns are consistent with observations from hospital, riverine, and open drainage settings across India (Gawande et al., 2025; Madhukar et al., 2024), reflecting differences in selective pressures, including variation in antibiotic usage patterns, sanitation infrastructure, healthcare facility density, treatment processes, and agricultural practices between urban and rural environments. In rural settings, for example, open drains often receive mixed waste from humans, livestock, and small-scale industries, creating hotspots for microbial interaction and horizontal gene transfer (Madhukar et al., 2024). The detection of these subtle but consistent differences suggests that local infrastructure and waste stream composition can shape both microbial ecology and resistance gene circulation. This has potential implications for pathogen and AMR transmission, as rural systems may promote more frequent microbial exchange across human, animal, and environmental reservoirs, whereas urban systems may reflect more anthropogenically driven selective pressures such as antibiotic residues and higher-density sewage inputs. The subtle but statistically significant urban–rural differences observed in microbial community structure and resistome composition merit careful interpretation. Urban catchments in Nagpur are characterized by higher population density, greater concentration of healthcare facilities (including tertiary hospitals), more extensive sewerage networks, and potentially higher burdens of pharmaceutical residues. These factors may create selective environments favoring particular resistance profiles and pathogen distributions. In contrast, rural catchments typically feature lower population density, limited wastewater treatment infrastructure, greater agricultural activity, more direct animal contact, and reliance on open drainage systems or surface water bodies. Such environments may facilitate different patterns of microbial exchange and horizontal gene transfer, particularly at the human–animal–environment interface. Similar urban–rural gradients in wastewater resistomes have been reported from other Indian regions (Diwan et al., 2018; Lamba et al., 2017; Marathe et al., 2017) and comparable LMIC settings (Ekwanzala et al., 2018; Auma et al., 2025; Nadimpalli et al., 2018), suggesting that local infrastructure, sanitation access, and anthropogenic activities are important drivers of geographic variation in AMR gene circulation (Collignon et al., 2018; Larsson and Flach, 2022; Van Boeckel et al., 2019). However, the overlapping nature of our ordination results indicates that these differences are subtle rather than categorical, likely reflecting the complex mosaic of influences operating across both urban and rural catchments in a rapidly developing region.

Our detection of 606 unique ARGs represents a markedly higher diversity than previously reported in Indian wastewater studies. For comparison, metagenomic surveys of Indian river sediments typically reported 50–150 unique ARGs (Singh et al., 2025; Gawande et al., 2025; Rout et al., 2023), while open drain studies from Hyderabad identified 89 ARGs and 287 antimicrobial resistance ontology (ARO) terms (Madhukar et al., 2024). This higher ARG diversity may reflect several factors: (1) deeper sequencing coverage in our study enabling detection of low-abundance genes; (2) use of the comprehensive MEGARes v3.0 database for ARG annotation; (3) the composite sampling strategy capturing resistance gene diversity across multiple sites; and (4) genuine differences in resistome complexity related to local antibiotic usage patterns, wastewater treatment infrastructure, and environmental selection pressures in central India. Importantly, the relative abundance patterns we observed, dominance of efflux pump genes and macrolide resistance determinants, align with reports from Hyderabad wastewater, where 23S rRNA mutations conferring macrolide resistance were similarly prevalent (Madhukar et al., 2024), suggesting consistency in certain resistance profiles across Indian urban centers despite differences in absolute ARG diversity.

Macrolides—classified as critically important by the WHO—are widely used in Indian clinical practice and agriculture (Chakraborty et al., 2024; Nair et al., 2010), often available over the counter and employed as poultry growth promoters (Hennessey et al., 2025). These practices likely drive the elevated levels of MLS resistance genes observed. ARG classes conferring resistance to aminoglycosides and tetracyclines were also frequently detected, consistent with patterns reported in hospital wastewater and major Indian rivers (Marathe et al., 2019; Rout et al., 2023; Sharma et al., 2024). Such findings underscore the need for environmental AMR surveillance that is contextualized to the location to inform national strategies and dissemination and implementation across countries of wide geography and environments. Phenotypic surveillance in four Indian cities, including Nagpur, has shown high bacterial resistance rates to erythromycin, tetracycline, vancomycin, ofloxacin, cefixime, and ampicillin (47–71%, Kapley et al., 2023), supporting our genomic findings.

The distinct ARG-bacterial genus correlation profiles suggest genus-specific resistome signatures that may reflect both intrinsic resistance mechanisms and patterns of acquired resistance via horizontal gene transfer. Wastewater environments represent recognized hotspots for horizontal gene transfer due to high microbial cell density, co-occurrence of diverse bacterial taxa, presence of selective agents (residual antibiotics, heavy metals, and biocides), and abundance of mobile genetic elements including plasmids, integrons, and transposons (Bengtsson-Palme et al., 2018; Guo et al., 2017). Recent metagenomic and genomic studies have demonstrated extensive ARG mobilization on plasmids recovered from wastewater-associated *Pseudomonas*, *Acinetobacter,* and *Aeromonas* isolates, supporting the interpretation that observed correlations reflect both genus-specific resistance profiles and ongoing horizontal gene transfer dynamics (Karkman et al., 2019; Ludden et al., 2019; Pärnänen et al., 2019; Petersen et al., 2019; Smalla et al., 2018). The strong correlation between copper resistance operons and efflux pumps in *Pseudomonas* is particularly noteworthy, as co-selection by heavy metals has been implicated in maintaining antibiotic resistance genes even in the absence of direct antibiotic pressure (Baker-Austin et al., 2006; Pal et al., 2015). This co-selection mechanism may be especially relevant in wastewater environments where both antibiotics and metals are present at sub-inhibitory concentrations. The strong associations between specific genera and ARG classes are consistent with other known resistance mechanisms: *Acinetobacter* species frequently harbor *blaOXA* carbapenemases and trimethoprim resistance determinants (Hamidian and Nigro, 2019; Royer et al., 2015); and *Aeromonas* species have been increasingly recognized as reservoirs of mobile resistance elements, including extended-spectrum beta-lactamases, tetracycline resistance genes, and sulphonamide resistance determinants (Igbinosa et al., 2012; Neil et al., 2024). These relationships indicate that the resistome structure in both urban and rural wastewater is shaped not only by the taxonomic composition of the microbial community but also by the horizontal transfer of ARGs and co-selection under complex environmental pressures.

We identified 13 of the 33 WHO priority bacterial pathogens responsible for 13.6% of all global deaths in 2019 (Ikuta et al., 2022; Naghavi et al., 2024), though only seven were detected in more than 10% of samples. Despite *Pseudomonas* prevalence at the genus level, *P. aeruginosa* was detected in only 6 of 138 samples, possibly due to strain-level detection limitations or low relative abundance below sequencing thresholds. While targeted approaches such as qPCR may offer greater sensitivity for specific pathogens (Ahmed et al., 2020; Maal-Bared et al., 2023), our findings illustrate that DNA metagenomics can concurrently capture a wide diversity of clinically relevant bacteria directly from wastewater (Brunfield et al., 2020; Hendriksen et al., 2019; Ng et al., 2019). This breadth underscores its value for characterizing subclinical and environmental circulation of multiple pathogens in parallel, providing complementary insights to targeted surveillance methods (Ng et al., 2019; Sims and Kasprzyk-Hordern, 2020).

The public health implications of our findings are substantial. Detection of high-priority resistance determinants, including *blaNDM* carbapenemases, *blaKPC* carbapenemases, and *mcr*-mediated colistin resistance genes in community wastewater, indicates circulation of last-resort antibiotic resistance mechanisms beyond clinical settings. While the relative abundance of these genes was low (2–3% of samples), their presence in environmental wastewater suggests potential for community transmission and highlights the importance of strengthening antimicrobial stewardship, infection prevention and control measures, and wastewater treatment infrastructure. The detection of the WHO priority pathogens, including members of the ESKAPE group, further underscores the value of wastewater-based surveillance as a complement to clinical microbiology systems. In resource-constrained settings where routine diagnostic capacity may be limited, wastewater metagenomics can provide cost-effective population-level surveillance of pathogen circulation and resistance trends, enabling early detection of emerging threats and informing public health interventions. Integration of wastewater surveillance data with clinical isolate databases, antimicrobial consumption data, and healthcare facility reports would create a more comprehensive AMR monitoring framework aligned with the WHO Global Action Plan on AMR and One Health principles (World Health Organization, 2015).

Our study has some limitations. First, we did not include phenotypic validation through culture-based isolation or antimicrobial susceptibility testing with clinically relevant pathogens (e.g., *Pseudomonas*, *Acinetobacter*, and *Aeromonas*). While metagenomic detection of resistance genes provides insight into genetic potential, it does not confirm functional expression or phenotypic resistance in viable isolates. Integration of culture-dependent approaches and antibiotic susceptibility testing in future studies would strengthen confidence in the clinical relevance of detected resistance determinants and establish genotype–phenotype links. Second, integration with clinical datasets was not possible as such data were not available. Aligning pathogen and ARG profiles from patients and wastewater would confirm the utility of wastewater as a proxy for population-level AMR surveillance. Thirdly, sample pooling—while enabling board geographical coverage—may mask fine-scale variation within individual sites. Additionally, sampling was conducted over a 3-month period (February–April 2021) during the dry season, and therefore, our findings may not capture seasonal variations in microbial composition or resistome dynamics that could occur during monsoon or winter periods. Longitudinal monitoring with absolute gene quantification would enable risk assessment, particularly for high-priority ARGs, such as *blaNDM*, *blaKPC*, and *mcr*. Longitudinal studies spanning multiple seasons and incorporating higher-resolution spatial sampling would provide further insight into temporal and local-scale drivers of wastewater microbiome structure and AMR gene dynamics. Fourth, untreated wastewater represents mixed human, animal, agricultural, and industrial inputs, but source attribution remains unresolved. Physicochemical data (e.g., nutrient load, heavy metal content, organic matter, pH, and temperature) were not collected as part of this analysis, limiting our ability to mechanistically link environmental variables to observed microbial and resistome patterns. Incorporating host-associated markers, antibiotic residues, and comprehensive environmental metadata in future prospective studies would help clarify resistance drivers and refine the interpretation of geographic variation. Finally, this study focused on one district and bacterial ARGs only. Broader geographic coverage, inclusion of viral and fungal resistance, and embedding wastewater metagenomics into national AMR surveillance frameworks will be critical for policy translation under a One Health approach. Furthermore, our findings are derived from urban and rural catchments in Nagpur district, central India, and reflect the specific demographic, environmental, and infrastructural context of this region. While these results may offer insights relevant to comparable settings in India and other low- and middle-income countries, direct extrapolation to regions with markedly different sanitation infrastructure, antibiotic usage patterns, or environmental conditions should be undertaken with caution. Broader geographic replication and comparative metagenomic surveillance across diverse Indian states and LMICs would strengthen the generalizability of these observations.

These limitations are offset by key strengths. Our dataset of 138 composites from 422 samples is among the largest DNA metagenomic wastewater surveys in India, spanning both urban and rural catchments. Use of untargeted shotgun metagenomics enabled simultaneous profiling of 871 genera, 606 ARGs, and 13 clinically important pathogens, including high-priority determinants, such as *blaNDM*, *blaKPC*, and *mcr*. Importantly, it is one of the few studies to compare microbial and resistome structures across urban and rural wastewater in India, offering ecological and public health insights.

From a One Health perspective, untreated wastewater represents a critical convergence point for microbial exchange between humans, animals, and the environment. In India, high antibiotic consumption, unregulated drug sales, limited wastewater treatment, and extensive environmental mixing zones create conditions that favor resistance evolution and dissemination (Laxminarayan and Chaudhury, 2016; World Health Organization, 2015). Our findings from Nagpur illustrate how metagenomic analysis of wastewater can capture clinically relevant pathogens and ARGs across urban–rural settings, offering insights with relevance beyond the local context. Looking forward, planetary health considerations must be incorporated into AMR planning: climate change-driven floods, droughts, and heatwaves will increasingly disrupt wastewater infrastructure and reshape microbial dynamics, with potential to accelerate resistance emergence (van Bavel et al., 2024). Addressing this challenge requires integrated strategies that combine metagenomics-based environmental monitoring with strengthened antibiotic stewardship and the development of sustainable alternatives such as bacteriophage or probiotic interventions.

## Data Availability

The datasets presented in this study can be found in online repositories. The names of the repository/repositories and accession number(s) can be found in the article/Supplementary material.

## Supplementary material

Supplementary Table 1 Sample collection information of all samples from urban, rural and natural wastewater sites used in the project. This data includes date and time of sample collection, weather metrics, location, locality and any local amenities.

Supplementary Table 2 Summary file documenting all antimicrobial resistance genes in each individual sample used in the project. This includes alignment metrics and the number of reads and depth associated to each gene.
