# Supplementary material for: Metagenomic insights into the urban–rural variation of antimicrobial resistance and pathogen reservoirs in untreated wastewater from central India
Source: Front Microbiol. 2026 Feb 11;16:1722229. doi: 10.3389/fmicb.2025.1722229 (PMC12932555; doi:10.3389/fmicb.2025.1722229)
Supplement: Supplementary Table 1 — Sample collection information of all samples from urban, rural and natural wastewater sites used in the project. This data includes date and time of sample collection, weather metrics, location, locality and any local amenities. [file Data_Sheet_1.zip › Figure 1.docx]

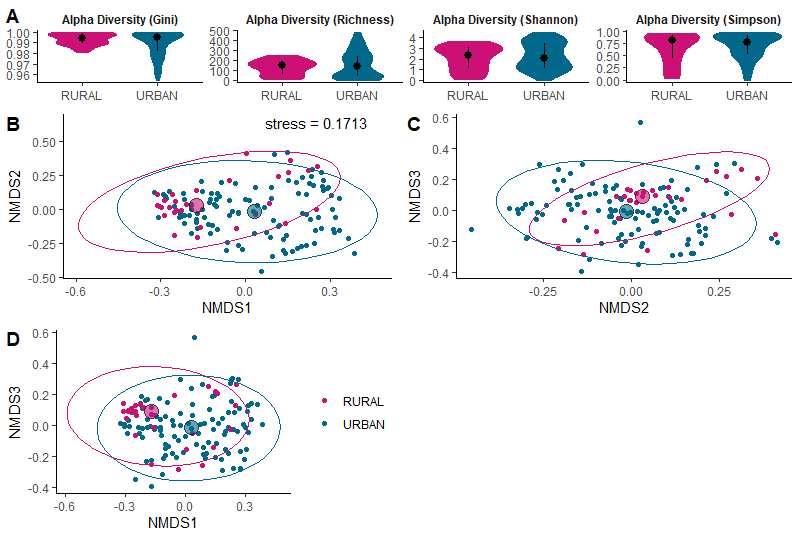


Supplementary Figure 1 (A) Violin plot showing four alpha diversity metrics, Gini, Richness, Shannon and Simpson for bacterial diversity of samples collected from rural and urban locations. Statistical differences tested using a Wilcoxon Ranked Sum test with Benjamini-Hochberg correction (p = 0.6). Black point shows median with 25^th^ and 75^th^ percentiles. (B) NMDS ordination plot showing NMDS1 and NMDS2 of Bray-Curtis dissimilarity of the bacterial composition of samples from urban and rural areas. Each point represents an individual sample; the separation of points reflects differences in the composition of samples. Larger points indicate the median values, and the ellipsis shows a 95% confidence interval of NMDS1 and NMDS2 for samples from each location. Stress indicates the stress value of the NMDS. (C) Scatter plot for NMDS2 and NMDS3. (D) Scatter plot for NMDS1 and NMDS3.
