# Supplementary material for: Metagenomic insights into the urban–rural variation of antimicrobial resistance and pathogen reservoirs in untreated wastewater from central India
Source: Front Microbiol. 2026 Feb 11;16:1722229. doi: 10.3389/fmicb.2025.1722229 (PMC12932555; doi:10.3389/fmicb.2025.1722229)
Supplement: Supplementary Table 1 — Sample collection information of all samples from urban, rural and natural wastewater sites used in the project. This data includes date and time of sample collection, weather metrics, location, locality and any local amenities. [file Data_Sheet_1.zip › Figure 2.docx]

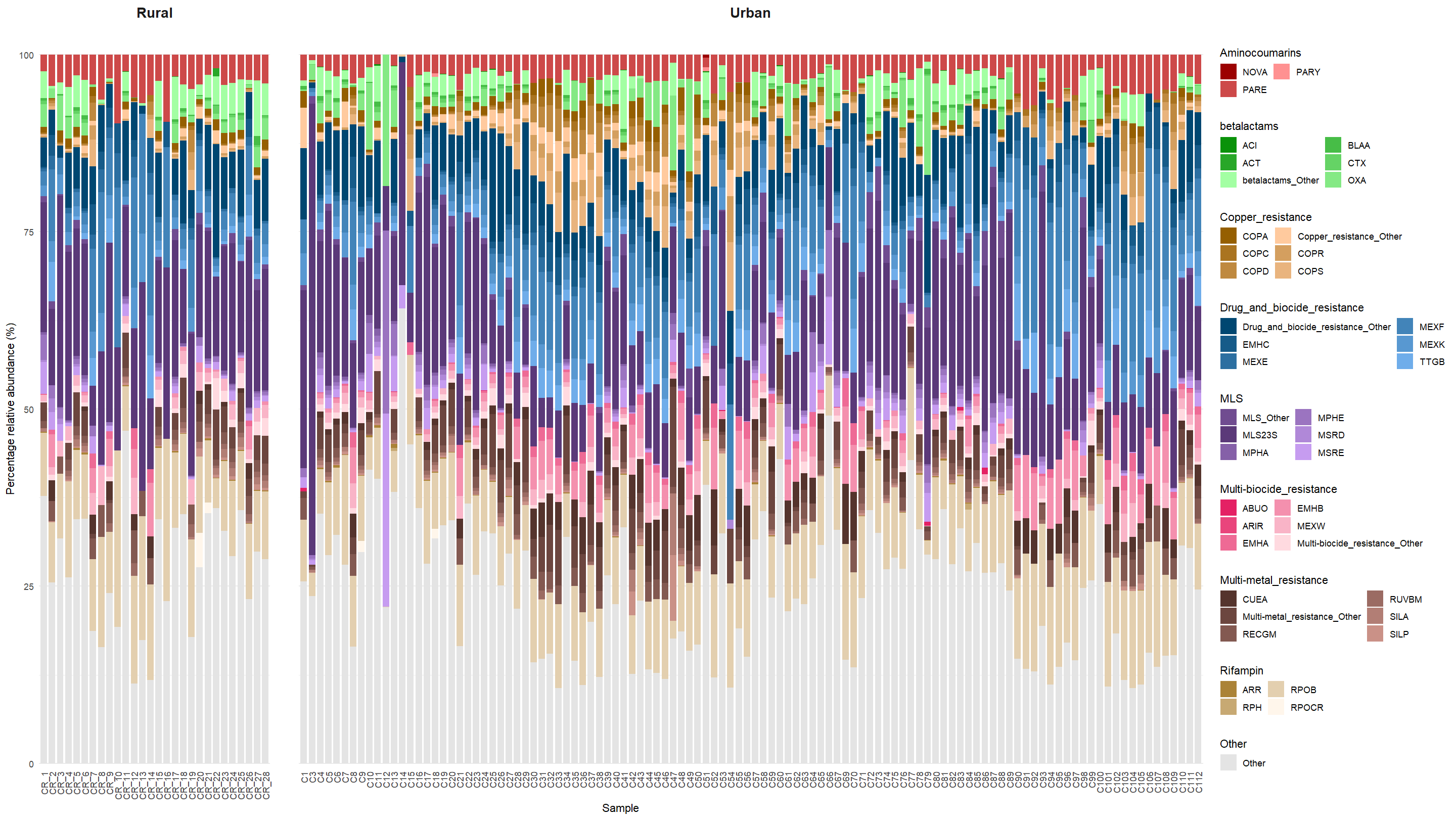


Supplementary Figure 2 Relative abundance of the eight antimicrobial resistance classes with the highest median abundance across all samples, showing the most abundant gene (by median abundance) within each class.
