# Supplementary material for: Metagenomic insights into the urban–rural variation of antimicrobial resistance and pathogen reservoirs in untreated wastewater from central India
Source: Front Microbiol. 2026 Feb 11;16:1722229. doi: 10.3389/fmicb.2025.1722229 (PMC12932555; doi:10.3389/fmicb.2025.1722229)
Supplement: Supplementary Table 1 — Sample collection information of all samples from urban, rural and natural wastewater sites used in the project. This data includes date and time of sample collection, weather metrics, location, locality and any local amenities. [file Data_Sheet_1.zip › Table 3.docx]

| Species | Median relative abundance (%) | No. samples detected |
| --- | --- | --- |
| *Acinetobacter baumannii* | < 1 | 23 |
| *Aeromonas* spp | 0.469 (3.02) | 112 |
| *Citrobacter* spp | < 1 | 15 |
| *Enterobacter* spp | 0 (0.0487) | 61 |
| *Escherichia coli* | 0.014 (0.210) | 85 |
| *Klebsiella pneumoniae* | 0.00604 (0.0964) | 73 |
| Other *Klebsiella* spp | < 1 | 34 |
| *Enterococcus faecium* | < 1 | 2 |
| *Morganella* spp | < 1 | 1 |
| Other *Enterococcus* spp | < 1 | 10 |
| *Providencia* spp | < 1 | 5 |
| *Pseudomonas aeruginosa* | < 1 | 6 |
| *Vibrio cholerae* | < 1 | 1 |

Supplementary Table 3 showing the median relative abundance (%) and interquartile range of clinically important pathogens identified in at least 10% of all samples.
